# TET1 Directs Chondrogenic Differentiation by Regulating SOX9 Dependent Activation of *Col2a1* and *Acan* In Vitro

**DOI:** 10.1002/jbm4.10383

**Published:** 2020-06-26

**Authors:** Piera Smeriglio, Fiorella Carla Grandi, Sarah Elizabeth Brook Taylor, Antoine Zalc, Nidhi Bhutani

**Affiliations:** ^1^ Department of Orthopaedic Surgery Stanford University School of Medicine Stanford CA USA; ^2^ Cancer Biology Program Stanford University School of Medicine Stanford CA USA; ^3^ Department of Chemical and Systems Biology Stanford University School of Medicine Stanford CA USA

**Keywords:** 5‐HYDROXYMETHYLCYTOSINE (5hmC), CHONDROGENESIS, EMBRYONIC CARTILAGE DEVELOPMENT, EPIGENETICS, METHYLATION, SKELETAL DEVELOPMENT, SOX9, TEN‐ELEVEN‐TRANSLOCATION ENZYMES (TET), TET1

## Abstract

Skeletal development is a tightly orchestrated process in which cartilage and bone differentiation are intricately intertwined. Recent studies have highlighted the contribution of epigenetic modifications and their writers to skeletal development. Methylated cytosine (5mC) can be oxidized to 5‐hydroxymethylcytosine (5hmC) by the Ten‐eleven‐translocation (TET) enzymes leading to demethylation. We have previously demonstrated that 5hmC is stably accumulated on lineage‐specific genes that are activated during in vitro chondrogenesis in the ATDC5 chondroprogenitors. Knockdown (KD) of *Tet1* via short‐hairpin RNAs blocked ATDC5 chondrogenic differentiation. Here, we aimed to provide the mechanistic basis for TET1 function during ATDC5 differentiation. Transcriptomic analysis of *Tet1* KD cells demonstrated that 54% of downregulated genes were SOX9 targets, suggesting a role for TET1 in mediating activation of a subset of the SOX9 target genes. Using genome‐wide mapping of 5hmC during ATDC5 differentiation, we found that 5hmC is preferentially accumulated at chondrocyte‐specific class II binding sites for SOX9, as compared with the tissue‐agnostic class I sites. Specifically, we find that SOX9 is unable to bind to *Col2a1* and *Acan* after *Tet1* KD, despite no changes in SOX9 levels. Finally, we compared this KD scenario with the genetic loss of TET1 in the growth plate using *Tet1* ^*−/−*^ embryos, which are approximately 10% smaller than their WT counterparts. In E17.5 *Tet1* ^*−/−*^ embryos, loss of SOX9 target gene expression is more modest than upon *Tet1* KD in vitro*.* Overall, our data suggest a role for TET1‐mediated 5hmC deposition in partly shaping an epigenome conducive for SOX9 function. © 2020 The Authors. *JBMR Plus* published by Wiley Periodicals, Inc. on behalf of American Society for Bone and Mineral Research.

## Introduction

Growth plate development involves the intricate coordination of transcription factors (TFs) and morphogens leading to condensation of the mesenchyme, deposition of extracellular matrix (ECM), and the eventual formation of bone.^(^ ^1^, ^2^ ^)^ Many of the genetic factors and signaling pathways involved in these processes have been identified. SOX9 is considered the master TF of chondrogenesis, playing a significant role in orchestrating endochondral ossification.^(^ ^3^ ^)^ Although TFs are important for transactivation of genes, it has become increasingly evident that the interaction of these TFs with epigenetic regulators is key to defining a particular lineage. A few of the epigenetic regulators known to contribute to chondrogenic lineage specification have been investigated, including the HDACs, DNMT3B, UHRF1, and the TET proteins.^(^ ^4^, ^5^, ^6^, ^7^, ^8^, ^9^, ^10^ ^)^ However, how these epigenetic regulators interact with SOX9 to set the epigenome up for chondrogenic lineage specification is largely unknown.

Cytosine modifications have established roles in development, cellular differentiation, and disease.^(^ ^11^, ^12^, ^13^ ^)^ Among these, 5‐methylcytosine (5mC) is the most widely studied in mammals, with known functions in X‐chromosome inactivation, imprinting, silencing of transposons, and gene transcription.^(^ ^12^, ^13^ ^)^ Recently, it was discovered that 5mC can be oxidized to 5‐hydroxymethylcytosine (5hmC), 5‐formylcytosine (5fC), and 5‐carboxylcytosine (5caC) iteratively by the Ten‐Eleven‐Translocation (TET) family of enzymes.^(^ ^14^ ^)^ The 5fC/5caC modifications can be targeted by DNA repair enzymes, such as thymine DNA glycosylase (TDG), to generate unmethylated cytosines or undergo replicative dilution, presenting two pathways to DNA demethylation.^(^ ^15^ ^)^ Intriguingly, 5hmC is stably accumulated in several tissues on enhancers^(^ ^16^ ^)^ and gene bodies of transcriptionally active genes.^(^ ^8^, ^17^, ^18^ ^)^ Our previous work has highlighted the accumulation of 5hmC in the ATDC5 progenitor cell line,^(^ ^8^ ^)^ as well as changes in the 5hmC landscape in osteoarthritic chondrocytes.^(^ ^19^, ^20^, ^21^ ^)^

Multiple loss‐of‐function studies highlight the importance of the TET enzymes for development.^(^ ^22^, ^23^, ^24^, ^25^ ^)^ Because of the TETs' catalytic and noncatalytic roles, it has been difficult to establish which of these developmental phenotypes are direct consequences of the loss of 5hmC. We have previously shown that 5hmC is stably accumulated on activated, lineage‐specific genes in differentiated chondrocytes and that knockdown (KD) of *Tet1* results in impaired chondrogenesis.^(^ ^8^ ^)^ A triple KO of *Tet1, Tet2*, and *Tet3* in mouse embryonic stem cells makes them lose differentiation potential toward mesodermal lineages, including cartilage and bone, suggesting the importance of 5hmC in skeletal development.^(^ ^26^ ^)^ Initial reports of the *Tet1* ^*−/−*^ mouse phenotype, by two different groups, demonstrated that mutants were smaller and weighed less than their WT counterparts.^(^ ^22^, ^27^, ^28^ ^)^ In contrast, the *Tet2* ^*−/−*^ mice have no skeletal phenotype^(^ ^24^, ^29^, ^30^ ^)^ and *Tet3* ^*−/−*^ are embryonic lethal.^(^ ^23^ ^)^ Given these compelling data for the role of TET1 in mesodermal development, we sought to further understand the role of TET1 in chondrogenesis. Here, we report that KD of *Tet1* impairs the differentiation of ATDC5 cells, partially through a loss of SOX9 binding at the critical lineage genes *Col2a1* and *Acan*. These effects are also observed in the growth plate of *Tet1* ^*−/−*^ embryos, albeit to a milder degree.

## Materials and Methods

### In vitro chondrogenic differentiation

ATDC5 progenitor cells were maintained in DMEM/F12, 5% FBS, 2mM/L glutamine, 1x antibiotic‐antimycotic (Fisher Scientific, Pittsburgh, PA, USA), and 12.5 μg/mL ascorbic acid (Eastman Chemical Company, Kingsport, TN, USA) in an undifferentiated state. Progenitors were differentiated to chondrocytes in basal media supplemented with 1× insulin‐transferrin‐selenite (ITS; Invitrogen, Carlsbad, CA, USA) for 15 to 20 days. Media was refreshed every 3 to 4 days. Cells were harvested at designated time points (day 0, 5, 10, or 15) with 0.5% trypsin/EDTA.

### Lentiviral shRNA transduction

Recombinant lentiviruses encoding shRNA for nontarget (NT) (SHC016‐1EA), Tet1(TRCN0000341917‐Tet1Sh1, TRCN0000341847‐Tet1Sh2, and TRCN0000341850‐Tet1Sh3; Sigma‐Aldrich, St. Louis, MO, USA) were produced with standard methods by cotransfection of pLKO.1 shRNA and packaging vectors in HEK293T cells. Cells were allowed to produce virus for 72 hours, after which it was harvested and concentrated with lenti‐X concentrator (TaKaRa Bio, Otsu, Japan) and used to infected ATDC5 cells overnight. shRNA‐Transduced ATDC5 cells were selected 48 hours posttransduction with 2 μg/mL of puromycin for 48 hours before differentiation in ITS. Before beginning differentiation, each batch of ATDC5 KD was checked for specific KD of *Tet1* (and not *Tet2* or *Tet3*).

### Protein immunoblots

Total protein content was extracted from progenitor cells using radioimmunoprecipitation assay lysis buffer (Thermo Fisher Scientific, Waltham, MA, USA). Blots were blocked for 2 hours in blocker/diluent buffer (Invitrogen), probed overnight with rabbit anti‐TET1 (1/200; Millipore, Billerica, MA, USA), SOX9 (1/100; Santa Cruz Biotechnology, Santa Cruz, CA, USA), or rabbit anti‐GAPDH (1/1000; Cell Signaling Technology, Beverly, MA, USA). After incubation with the appropriate peroxidase‐labeled secondary antibody (1/5000; Santa Cruz Biotechnology), blots were visualized using Luminata Forte HRP substrate (Millipore) and quantified using FiJi software (NIH, Bethesda, MD, USA).

### ELISA for 5hmC levels

To detect global 5hmC levels in *Tet1 KD*, we used the MethylFlash Hydroxymethylated DNA Quantification Kit Colorimetric Assay (Epigentek, Farmingdale, NY, USA) as per the manufacturer's directions. DNA was extracted using the DNAeasy Kit (QIAGEN, Valencia, CA, USA); 200 ng was used for each sample. Samples were repeated in technical duplicates.

### DNA immunoblots

Total genomic DNA was extracted using the DNeasy Kit (QIAGEN), denatured (0.4M sodium hydroxide, 10mM EDTA at 100°C for 10 min), and then neutralized (6.6M cold ammonium acetate, pH 7). We applied 200 ng of DNA to a prewet Amersham Hybond‐N+ membrane (GE Healthcare Life Sciences). The membrane was blocked and incubated in primary antibody overnight (anti‐5hmC and anti‐5mC 1/200; Active Motif, Inc., Carlsbad, CA, USA), followed by the appropriate peroxidase‐labeled secondary antibody (1/5000; Santa Cruz Biotechnology). Blots were visualized using Luminata Forte HRP substrate (Millipore), and quantified using ImageJ software (NIH; https://imagej.nih.gov/ij/).

### Glycosaminoglycan staining

Glycosaminoglycan (GAG) staining was carried out using the Alcian Blue Staining Kit (Lifeline Cell Technology, Frederick, MD, USA) on ATDC5 cells that had been fixed with 4% paraformaldehyde in PBS.

### Microarray analysis

Total RNA was extracted as previously described from cells using the RNeasy Kit (QIAGEN), and RNA integrity was checked using the bioanalyzer. Samples for NT controls, *Tet1 Sh1* and *Tet1 Sh2*, were applied to Mouse Gene 1.0 ST Arrays (Affymetrix, Santa Clara, CA, USA). Normalization, comparison of gene expression values, filtering of significant expression probes, and clustering analysis of expression values were done within dChip^(^ ^31^ ^)^ as described in the manual. The default model‐based expression method (with invariant set probe selection method and running median smoothing method) was used for the normalization step. To analyze the changes in gene expression with *Tet1* KD, the NTt sample was set as the baseline (B) array and the *Tet1* KD replicates as the experimental (E) arrays. To identify probes that showed differential expression, three filtering criteria were put in place: (i) 2.5‐fold change in the B/E or E/B arrays; (ii) 100 intensity units difference between B and E arrays; and (iii) selection of the lower 90% confidence bound. Prior to clustering analysis, filtering was performed; probes were extracted based on consistent levels of intensity in at least two arrays (2/4 = 50% of arrays) and an intensity of at least 150; 1152 of 35512 probe sets satisfied this filtering criteria. Clustering was performed using default settings. Differentially expressed genes are listed in Supplementary Table S1. Data are deposited in the Gene Expression Omnibus (GEO; NIH, https://www.ncbi.nlm.nih.gov/geo/) under series GSE105122.

### Gene expression analyses

Total RNA was extracted from tissues as previously described and from cells using the RNeasy Kit (QIAGEN), and reverse‐transcribed with a high capacity cDNA RT Kit (Applied Biosystems, Foster City, CA, USA). qPCR was performed using TaqMan probes (Applied Biosystems, Foster City CA) for *Tet1* (Mm01169087_m1), *Tet2* (Mm00524395_m1), *Tet3* (Mm00805756_m1), *Sox9* (Mm00448840_m1), *Col2a1* (Mm01309565_m1), *Col10a1* (Mm00487041_m1), and *Acan* (Mm00545794_m1) with a universal mastermix (Applied Biosystems) using *Gapdh* (Mm99999915_g1) as an internal control. All analyses were performed using the ΔΔCT method and expression was normalized to *Gapdh*.

### Locus‐specific detection of 5hmC

Detection of 5hmC at a particular CCGG site was performed using an EpiMark 5hmC and 5mC Analysis Kit (New England BioLabs, Ipswich, MA, USA) as per the supplier's protocol. The EpiMark‐treated DNA was subjected to qPCR using site‐specific primers. The percentage of 5hmC was calculated using the EpiMark comparative cycle threshold (Ct) method. Primer sequences: *Sox9* Peak 1 (F: AAAGCGAAGCTTTGCAAGAA, R: AAGGTTGGCTAAGGGAGGAA) *Sox9* Region 2 (F: CTTTTCTCTTTGCGCCTCAC, R: TGGTTGCCAAGGTGTCATTA) *Acan* peak 1 (F: TCTGTCACCCATCTCCTTCC, R: TCCAGCCAGCGTCTAAGTTT), *Col2a1* peak 1 (F: ACAAGCGTCTCCAATCCATC, R: ACAGAGGGAGACCTGTGTGG), *Col2a1* peak 2 (F:TCTTTCGGGGAACTGTTTTG, R:CCTCTCCCACAATGCACAG).

### Profiling of hydroxymethylated and methylated DNA

Total DNA was extracted from ATDC5 cells and enriched for 5hmC using a biotin‐based streptavidin pull‐down technique (Hydroxymethyl Collector; Active Motif, Inc.) or for 5mC using a Methylated CpG Island Recovery‐Based Assay (MIRA; MethylCollector Ultra, Active Motif, Inc.), as per the manufacturer's guidelines. Libraries were prepared using 500 ng of 5hmC or 5mC enriched DNA using the NEBNext DNA Library Prep Master Mix Set for Illumina (New England BioLabs) and were sequenced on an Illumina HiSeq 2000 (Illumina, San Diego, CA, USA) with single end (1 × 50 base pair) reads. DNA was collected at day 0, day 10, and day 20 of differentiation.

### Analysis of methylated and hydroxymethylated DNA profiling

An iterative quality check using FASTQC (http://www.bioinformatics.babraham.ac.uk/projects/fastqc/) and filtering procedure using Cutadapt (http://code.google.com/p/cutadapt/) was performed to obtain good quality reads after trimming of the initial seven bases. The Burrows‐Wheeler Aligner, version 0.7.5a‐r405 (http://bio-bwa.sourceforge.net/)^(^ ^32^ ^)^ was used with default parameters to align the filtered and trimmed reads to the mouse reference genome (GRCm38/mm10; http://genome.UCSC.edu), and alignments with a MAPQ (Mapping Quality) score of >5 were used for downstream analyses. To identify regions that were gaining/losing 5mC/5hmC in chondrocytes compared with progenitors, MACS version 1.4.2 (http://liulab.dfci.harvard.edu/MACS/)^(^ ^33^ ^)^ was used to call peaks using default settings. The MEDIPS package])^(^ ^34^ ^)^ was used to assess the sequencing depth and coverage of 5mC and 5hmC across the genome. The saturation was calculated using default settings. First, redundant reads were removed. If some reads mapped from end‐to‐end perfectly to another read, only one of these reads (the representative) was considered. To find the saturation, the set of unique reads was then divided into two halves, A and B, of equal sizes. These two sets were then randomly divided into subsets of equal size (number of subsets determined by the default parameter, nit =10). In iterations, an increasing number of subsets from A and B was used to calculate the correlation between these subsets of A and B. As more subsets were used, the correlation became greater. To examine the reproducibility of the total set of available short reads, MEDIPS followed‐up with an estimation of saturation, the original data set was artificially doubled, then proceeds with the correlation calculation as before to ascertain whether the unique short reads would have captured the main pattern of 5hmC from the genome. A moderate‐to‐high correlation value for saturation indicated reproducibility. Data are deposited in GEO under series GSE105122.

### Peak annotation, motif analysis, and data visualization

Peaks were annotated using the annotatePeaks.pl program in HOMER (Hypergeometric Optimization of Motif EnRichment; http://homer.ucsd.edu/homer/index.html). Motif analysis for 5hmC deposition was performed using HOMER's findMotifsGenome.pl. 5hmC and 5mC reads were plotted using ngs.plot.^(^ ^35^ ^)^

### Cross‐referencing data to other published data sets

Differentially expressed genes were cross‐referenced against other published data sets. *SOX9 Peak analysis*

SOX9 ChIP‐ (chromatin immunoprecipitation‐) seq data were downloaded from GSE69109.^(^ ^36^ ^)^ The genes associated with SOX9 binding sites were then overlapped with genes from differential expression analysis. Note that the overlap here implies that sections of approximately 150 bp (the size of a 5hmC or SOX9 peak) overlap for at least one base pair or more.

*5hmC Peak analysis*

To determine if differentially expressed genes had 5hmC peaks in chondrocytes (day 20), we used our previously published data set.^(^ ^8^ ^)^ 5hmC peaks were intersected with differentially expressed genes using the intersect intervals tool on galaxy (https://usegalaxy.org/).^(^ ^37^ ^)^

*Enhancer and superenhancer analysis*

Coordinates for previously established enhancer and superenhancers locations in postnatal rib chondrocytes were taken from GSE69109^(^ ^36^ ^)^ and analyzed as above.

### Chromatin immunoprecipitation

shRNA‐Transduced ATDC5 cells (approximately 5 × 10 6) were cross‐linked with 1% formaldehyde (10 min at room temperature). ChIP assays were performed using the ChIP‐Express Enzymatic Kit (Active Motif, Inc.) according to the manufacturer's instructions. Briefly, chromatin was enzymatically sheared to an average size of approximately 300 bp and incubated with 2 μg of control IgG (Active Motif, Inc.) or antibody specific to SOX9 (Abcam, Cambridge, UK), and 25 μL of protein G magnetic beads overnight at 4°C. After reversal of cross‐linking and protein digestion with proteinase K, immunoprecipitated DNA was purified with the MiniElute PCR Purification Kit (QIAGEN), and PCR was performed using a SYBR Green ROX Master Mix (QIAGEN) according to the following parameters: enzyme activation, 95°C for 2 min, and then 40 cycles of 95°C for 15 s and 60°C for 1 min. EpiTect ChIP qPCR primers (QIAGEN) for the putative SOX9 binding site were used to amplify the target enhancer region within the *Acan* (GPM10391229‐05 kb) and *Col2a1* (GPM1046416 + 03 kb) genes. Each probe represented a pool of primers that amplify many different amplicons around that region. For *Col2a1*, this is a region +3 kb from the transcription start site (TSS; Active Motif, Inc.) and for *Acan* this is a region –5 kb from the TSS. Both regions include previously described SOX9 binding sites.

### Generation of *Tet1*^*−/−*^ mice and defined stage embryos

All animal procedures were approved by the Stanford University Administrative Panel on Laboratory Animal Care (APLAC). *Tet1* ^*+/−*^ mice (Jackson Laboratory, Bar Harbor, ME, USA) were time‐mated and the females euthanized at 13.5 and 17.5 days postcoitus. Embryos were harvested and genotyped using PCR. Genotyping primers: WT forward: 5′‐TCAGGGAGCTCATGGAGACTA‐′, mutant forward: 5′‐AACTGATTCCCTTCGTGCAG‐3′, common reverse: 5′‐TTAAAGCATGGGTGGGAGTC‐3′.

### Whole‐skeleton staining

Embryos were dissected, fixed overnight in 95% ethanol, and stained with Alcian Blue. The stained embryos were then treated with 2% KOH (potassium hydroxide) for 24 hours and in 1% KOH/20% glycerol for 2 days to remove the excess dye. E17.5 embryos were additionally stained for bone with an overnight staining in Alizarin Red before the KOH treatment to remove excessive dyes.

### DNA and RNA extraction from embryonic samples

Cartilage from E17.5 hind limbs was dissected after removal of skin and bone and snap‐frozen in liquid nitrogen. Following manual grinding with mortar and pestle, the tissue was homogenized with a tissue raptor in Trizol buffer (Life Technologies Inc., Grand Island, NY, USA). A standard phenol‐chloroform protocol was then used for phase separation and DNA or RNA precipitation.

### RNA‐sequencing library preparation

RNA from embryos was utilized to make RNA‐sequencing libraries using TruSeq Stranded Total RNA Library Prep Kit with Ribo‐Zero (Illumina) according to the manufacturer's instructions. Before sequencing, libraries were quantified by Qubit fluorometric quantitation (Thermo Fisher Scientific) and by bioanalyzer (Agilent Genomics, Santa Clara, CA, USA). Only samples with a RNA integrity number (RIN) between 7 and 10 were used. Three *Tet1* ^*+/+*^ and *Tet1* ^*−/−*^ mutant samples were indexed and pooled into one lane. Sequencing was performed on the HiSeq2500 as paired‐end 100 base pair reads. On average, 45 million paired reads were obtained per sample. RNA‐sequencing data were deposited in GEO series GSE105122.

### Differential expression analysis

RNA‐sequencing quality was analyzed using FASTQC (http://www.bioinformatics.babraham.ac.uk/projects/fastqc/). Reads were trimmed using Trimmomatic.^(^ ^38^ ^)^ On average, 85% of the paired‐end reads were retained for downstream analysis. RNA‐sequencing analysis was done following the Tuxedo pipeline.^(^ ^39^ ^)^ Briefly, reads were mapped to the mm10 (http://genome.ucsc.edu/) genome using HISAT2.^(^ ^40^ ^)^ Transcripts were then called using Stringtie.^(^ ^41^ ^)^ Stringtie can call transcripts both from known GFF (generic feature format) files, which were downloaded from the University of California, Santa Cruz genome browser, and simultaneously build a de novo transcript library from the samples. Differential gene expression analysis was performed using Ballgown.^(^ ^42^ ^)^ For our downstream analysis, we focused on annotated transcripts with a greater than 2.5 change in expression with an adjusted *p* value of <0.05, although approximately 50% of the differentially expressed genes were not annotated in mm10. Pathway analysis on differentially expressed genes was performed using Enrichr^(^ ^43^, ^44^ ^)^ and STRING.^(^ ^45^ ^)^ Heat maps were generated using the Seaborn package for Python (https://pypi.python.org/pypi/seaborn). Supplementary Table S1 contains the genes used for differential expression analysis.

### Immunofluorescence and quantification

Immunofluorescence was performed as previously described.^(^ ^8^ ^)^ Briefly, embryonic tissue sections were permeabilized in methanol and heat‐treated with citric acid buffer for antigen retrieval before blocking and incubation with primary antibody overnight (anti‐Sox9 1:50, Santa Cruz Biotechnology; anti‐5hmC 1:100, Active Motif, Inc.). After secondary antibody incubation (Alexa 594 goat anti‐rabbit 1:250; Invitrogen) cellular DNA was counterstained with 4,6‐diamidino‐2‐phenylindole (Life Technologies). Quantification of images was performed using FiJi using the “measure” feature.

### Isolation of primary chondrocytes from embryonic growth plates for Western blot analysis and FACS

Limbs were isolated from E17.5 embryos. After removal of the skin and soft tissues, the femur was dislocated and translucent cartilage tissue was isolated from dark bone areas and placed in 1X PBS. The tissue was initially digested for 60 min with collagenase P (2 mg/mL in PBS; Roche, Basel, Switzerland) at 37°C with continuous shaking, followed by incubation with collagenase D (3 mg/mL dissolved in serum‐free Dulbecco's modified Eagle's medium) in a 37°C incubator for 5 hours. The digested tissue solution was filtered through a 70‐μm filter to eliminate undigested debris and then centrifuged. Cells were resuspended in complete medium (DMEM/F12, 10% FBS, 1% penicillin/streptomycin, 100‐mM/L glutamine, and 50‐μg/mL ascorbic acid, pH 7.1). To remove any contaminating fibroblasts, cultures were treated with 0.05% trypsin for 1 min after 24 hours and the media changed to remove the fibroblasts, while allowing the chondrocytes to remain attached. Cells were cultured for approximately 7 days until confluent and used at P1. The purity of the isolated cell populations was tested by FACS analysis after cell staining with the following antibodies: SOX9‐PE, CD200‐PerCP, and CD24‐FITC.

### Reduced representation 5hmC profiling, analysis, and calling of differentially hydroxymethylated CCGGs

Reduced representation 5hmC profiling (RRHP) analysis^(^ ^46^, ^47^ ^)^ was performed as previously described.^(^ ^21^ ^)^ Briefly, 1 μg of DNA was processed using the RRHP 5‐hmC Library Prep Kit from Zymo Research (Cat. D5450; Zymo Research, Irvine, CA, USA) according to the manufacturer's specifications. Library‐size selection was done using Agencourt AMPure Beads (A63881; Agencourt Bioscience Services, a division of Beckman Coulter Genomics, Danvers, MA, USA) at a 1.8× ratio. Libraries were indexed using NEBNext Multiplex Oligos Ultra Index Primers (Set 1, E7600S; New England BioLabs) and amplified for 15 cycles. Individual libraries were analyzed on the bioanalyzer and pooled to a final concentration of 8 to 12 picomolar (pM). A negative control library was generated by including a sample not treated with T4‐glucosyltransferase (T4 BGT). Pooled libraries were sequenced on the Illumina HiSeq 4000 as paired‐end 101 base‐pair reads. On average, 40 million paired reads were obtained per sample. Reads were trimmed using TrimGalore (https://github.com/FelixKrueger/TrimGalore) and mapped to the mm10 genome with HISAT2.^(^ ^40^ ^)^ SAMtools sort was used to sort sam files^(^ ^48^ ^)^ and BEDOPS bamtobed was used to create bed files.^(^ ^49^ ^)^ Differentially hydroxymethylated CCGGs were called using diffREPS with a window size of 200.^(^ ^50^ ^)^ Peaks with an adjusted *p* value of 0.05 were used for downstream analysis. MACS2 was also used to make bed files for peaks in TET1 WT and KO samples.^(^ ^33^ ^)^ Supplementary Table S4 contains a list of differentially hydroxymethylated sites. Data are deposited in GEO under accession number GSE105122.

## Results

### Knockdown of *Tet1* impairs chondrogenesis in ATDC5 cells

**Fig 1:**
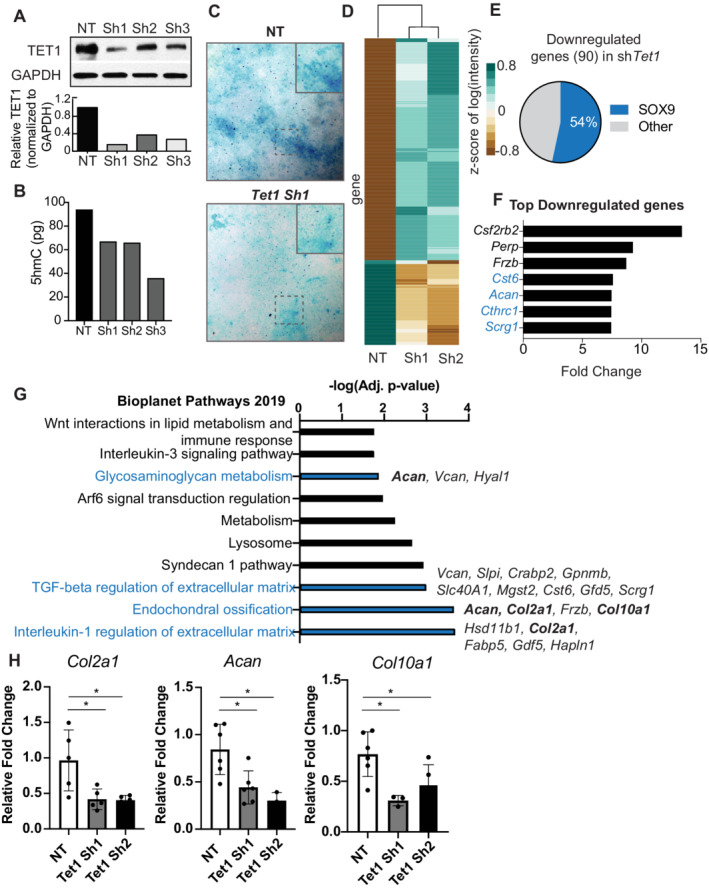
*Tet1* knockdown impairs chondrogenesis in ATDC5 cells. (*A*) Representative Western blot analysis for TET1 and GAPDH proteins in ATDC5 progenitor cells infected with control (nontarget [NT]) or *Tet1* shRNA (Sh1, Sh2, Sh3) at day 15 of differentiation. Quantification is represented as TET1 expression relative to GAPDH. (*B*) Quantification of global 5hmC (pg) in ATDC5 NT control and *Tet1* shRNA by ELISA at day 15 of differentiation. (*B*) Representative Alcian Blue staining for ATDC5 progenitors differentiated to chondrocytes in the presence (NT) or absence (Tet1Sh) of TET1 at day 15. (*D*) Heat map of differentially expressed genes in ATDC5 KD at day 15 from microarray analysis. Heat map is clustered based on the *Z* score of the standard log of probe intensity. (*E*) Pie chart of percent of the 90 downregulated genes that are SOX9 targets. SOX9 targets are defined from Ohba and colleagues.^36^ (*F*) Top downregulated genes from microarray upon *Tet1sh*. SOX9 target genes are highlighted in blue. (*G*) Top pathways associated with *Tet1* KD in ATDC5 cells, from Bioplanet curated pathways, made using Enrichr. Key extracellular matrix genes are bolded. (*H*) Real‐time PCR validation for *Col2a1*, *Acan*, and *Col10a1* in NT control and *Tet1sh* ATDC5 chondrocytes at day 15. Expression is normalized to *Gapdh* and fold‐change is relative to one of the NT controls. Data are represented as the mean ± SD. *p < 0.05 (Student's *t* test). *n* = 5 for each condition.

Our previous work characterized the accumulation of 5hmC during chondrocyte maturation in ATDC5 cells,^(^ ^8^ ^)^ a teratoma cell line that can be induced reproducibly and homogeneously toward chondrogenic differentiation.^(^ ^51^ ^)^ We observed this accumulation especially along the regulatory regions before the TSS and gene bodies of lineage specific genes.^(^ ^8^ ^)^ To investigate the effect of acute *Tet1* loss, and thus 5hmC, we utilized multiple short hairpin RNAs (shRNAs) to KD *Tet1* (Fig. 1*A*), with minimal effect on the other TET family paralogues, *Tet2* and 3 (Supplementary Fig. S1 *A*). This led to a 40% to 60% loss of total 5hmC deposition (Fig. 1*B*), which was stable throughout differentiation (Supplementary Fig. S1 *B*), although global methylation levels were not affected (Supplementary Fig. S1*B*).

Loss of *Tet1* resulted in impaired chondrogenesis with a decrease in the proteoglycan content that is deposited by differentiated chondrocytes (Fig. 1*C*). To better understand the gene programs controlled by TET1, we performed microarray analyses. NT control and *Tet1*‐deficient ATDC5 cells with two independent shRNAs were profiled after 15 days of differentiation (Fig. 1*D*, Supplementary Table S1). We observed 236 upregulated genes and 90 downregulated genes (Fig. 1*D*). When we cross‐referenced our differentially expressed genes with a previously published SOX9 ChIP‐seq data set,^(^ ^36^ ^)^ we observed that among the 90 genes that were downregulated in the *Tet1* KD cells, 54% were SOX9 targets (Fig. 1*E*). The downregulated Sox9 targets included *Cst6, Acan, Cthrc1*, and *Scrg1* (Fig. 1*F*), although the expression of *Sox9* or its known partner proteins (such as *Sox5, Sox6, Runx2,* and *Twist1*) were not affected (Supplementary Table S1). In agreement with the observed phenotype, a number of genes important for chondrogenic fate relating to GAG metabolism, TGF‐β regulation of ECM, and endochondral ossification were downregulated, including, *Vcan, Col2a1, Col10a1*, and *Hapln1* (Fig. 1*G*). Intriguingly, 72% of the genes differentially expressed after *Tet1KD* were upregulated (Fig. 1*D*). This likely reflects TET1ʼs role as a repressor via its interaction with the PRC2 complex.^(^ ^14^, ^52^ ^)^ Among the pathways enriched in these upregulated genes were interferon responses and leptin signaling (Supplementary Fig. S1*C*). In contrast to the downregulated genes, only 28% of the genes were SOX9 targets (Supplementary Fig. S1*D*). These may represent targets of SOX9ʼs repressive abilities,^(^ ^53^ ^)^ although this function is less understood.

As 5hmC deposition is generally associated with gene activation,^(^ ^54^ ^)^ we decided to focus on the downregulated genes. We validated three of the downregulated targets, *Col2a1*, *Col10a1*, and *Acan* using qPCR (Fig. 1*H*) on ATDC5 cells after 15 days of differentiation. As SOX9 is one of the major TFs controlling chondrogenic fate^(^ ^3^ ^)^—and is involved in the regulation of *Col2a1*, *Col10a1*, and *Acan—*we hypothesized that impairment in SOX9 expression or function might cause the observed block in differentiation.

### *Tet1* knockdown does not directly regulate SOX9 gene expression

**Fig 2:**
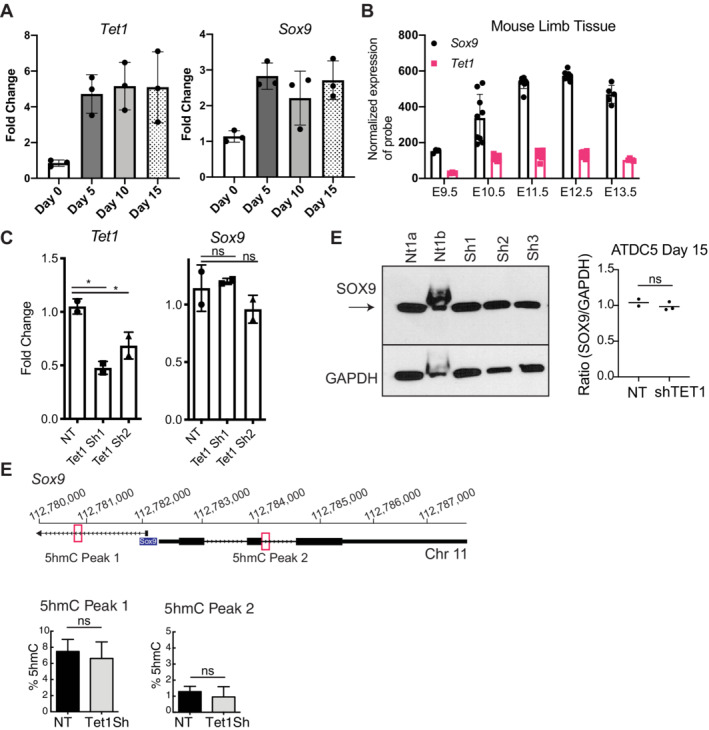
*Tet1* KD does not regulate SOX9 expression in ATDC5 cells. (*A*) Real‐time PCR of *Tet1* and *Sox9* expression during a time‐course ATDC5 differentiation. Expression is normalized to *Gapdh* and fold‐change is relative to one of the day 0 controls. *n* = 3 for each timepoint. (*B*) Microarray data from limbs collected during mouse development, for probes corresponding to *Tet1* and *Sox9*. Data reanalyzed from Taher and colleagues^(^ ^75^ ^)^; each point represents the normalized probe intensity for a biological replicate. (*C*) Real‐time PCR gene expression analysis of *Tet1* and *Sox9* in nontarget (NT) control and *Tet1sh* ATDC5 chondrocytes at day 15 of differentiation. Expression is normalized to *Gapdh* and fold‐change is relative to one of the NT controls. Data are represented as the mean ± SD. ns = not significant. *p = 0.01 (sh1) and 0.04 (sh2; one‐way ANOVA with multiple comparisons). *n* = 2 for each condition. (*D*) Western blot analysis for SOX9 (arrow designates band) and GAPDH proteins in ATDC5 cells at day 15 infected with NT or *Tet1* shRNA (Sh1, Sh2). NT shRNAs represent two technical replicates (independent infections) with the same NT shRNA. Quantification is represented as the intensity of the TET1 band relative to the GAPDH band on the graph to the right. ns = not significant (Student's *t* test). (*E*) Percentages of 5hmC at locus‐specific CCGG sites in *Sox9*. Location of the tested 5hmC peaks is designated on the diagram at the top. Values are expressed as the mean ± SD in *Tet1sh* and NT control (*n* = 3) ns = not significant (Student's *t* test).

To understand the relationship between TET1 and SOX9, we first began by assaying the expression of *Sox9* and *Tet1* mRNA during ATDC5 differentiation. We observed the expected increase in *Sox9* expression during early chondrogenic induction, ie, at day 5, with *Tet1* expression also increasing concomitantly in a similar pattern (Fig. 2*A*). These dynamics mirrored what is observed in the mouse limb (Fig. 2*B*) and in human embryonic stem cells (hESCs) induced toward the mesodermal fate (Supplementary Fig. S1 *E*). Because of these expression patterns and the previously observed accumulation of 5hmC on the SOX9 gene during ATDC5 differentiation,^(^ ^8^ ^)^ we initially hypothesized that *Sox9* might be a direct or indirect transcriptional target of TET1. However, *Tet1* KD did not result in a robust downregulation of *Sox9* mRNA prior to differentiation at day 0 (Supplementary Fig. S1*F*), or after differentiation at day 15 (Fig. 2*C*), as assayed by real‐time PCR or microarray (Supplementary Table S1). To corroborate this mRNA data, we performed Western blot analyses of SOX9 protein levels on day 15 chondrocytes after *Tet1* KD, and did not observe any change in SOX9 protein levels either (Fig. 2*D*). These data are in contrast to reports of the role of TET1 in intestinal stem cell (ISC) differentiation, in which TET1 was found to control many WNT target genes including SOX9 in a 5hmC‐dependent manner.^(^ ^55^ ^)^ In ISCs, it was observed that TET1‐mediated 5hmC deposition on the *Sox9* promoter regulates its expression. In our previous characterization of global ATDC5 5hmC profiles at day 20 of differentiation, we observed one prominent peak on the *Sox9* TSS and one near the gene body.^(^ ^8^ ^)^ We assayed the effect of *Tet1* KD on 5hmC deposition at these two CCGG sites in *Sox9*, but observed no decrease in 5hmC (Fig. 2*E*). Collectively, these observations suggest that in chondrocytes, as opposed to ISCs, TET1‐mediated 5hmC does not regulate *Sox9* gene expression. It remains possible, however, that *Tet1* KD may have other indirect effects on SOX9 protein function in the ATDC5 system, which were not exhaustively assayed here.

### 5hmC is deposited on a subset of SOX9 target genes

**Fig 3:**
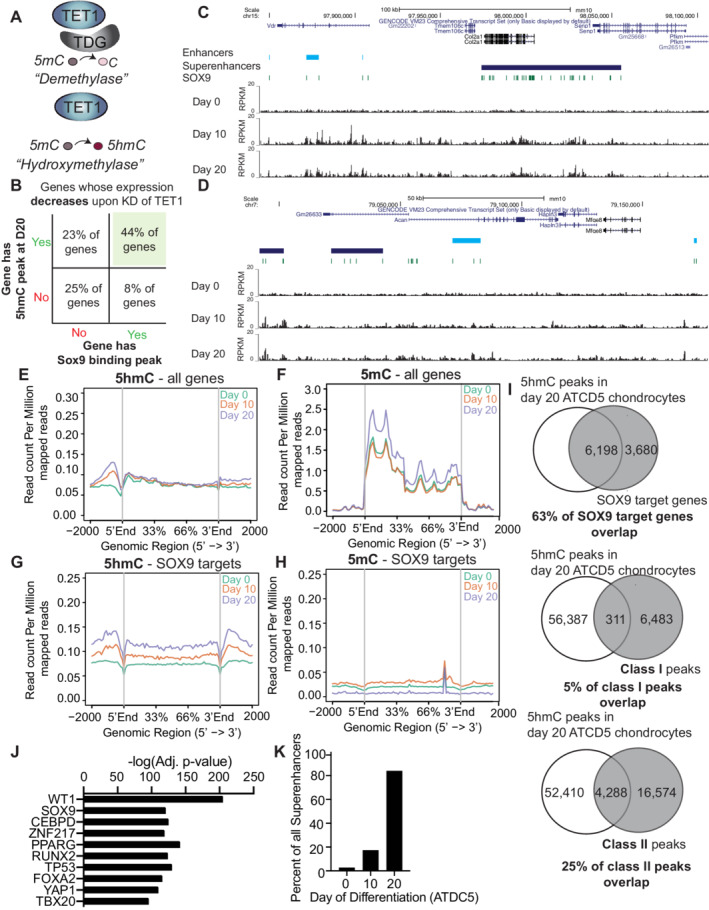
A subset of SOX9 target genes gain 5hmC deposition during chondrogenesis. (*A*) Schematic of two possible roles for TET enzymes, one as a demethylase primarily converting existing 5mC to unmethylated C and one as a hydroxymethylase, converting 5mC to stable 5hmC. (*B*) Percent of genes whose expression is decreased in *Tet1KD*, which have SOX9 peaks and 5hmc peaks. (*C,D*) Representation of *Acan* (*C*) and *Col2a1* (*D*) genes with SOX9 binding sites, enhancers, and superenhancers (top) and 5hmC profile (bottom) in day 0, 10, and day 20 differentiated ATDC5 chondrocytes. 5hmC peaks are in Reads Per Kilobase Million (RPKM). (*E,F*) Composite profiles of the 5hmC and 5mC distribution on all annotated genes during differentiation from day 0, 10, and 20 ATDC5 cells over 5′‐3′ gene length ± 2000 bp. (*G,H*) Composite profiles of the 5mC and 5hmC distribution on SOX9 target genes during differentiation from day 0, 10, and 20 cells averaged over 5′‐3′ gene length ± 2000 bp. (*I*) Percent of all SOX9 genes that contain a 5hmC peak in day 20 ATDC5 chondrocytes (top). This is then further broken down into SOX9 peaks (either class 1 or class 2), which directly overlap with a 5hmC peak. Class I or class II peak locations from Frazee and colleagues^42^. (*J*) Transcription factors (TFs) upstream of the SOX9 target genes that contain 5hmC peaks in day 20 ATDC5 chondrocytes. (*K*) Percent of superenhancers that overlap with 5hmC peaks throughout ATDC5 differentiation.

To understand how TET1 loss affects SOX9 targets, we next sought to investigate the relationship between 5mC, 5hmC, and these targets. In particular, we were interested in determining if TET1 was acting predominantly as a hydroxymethylase or as a DNA demethylase in this context (Fig. 3*A*). Although the canonical SOX9 motif does not contain any CG dinucleotides,^(^ ^36^ ^)^ the typical sites of mammalian cytosine modification, it was possible that SOX9 target genes needed to be demethylated prior to activation. Indeed, among the 90 downregulated genes in ATDC5 cells, we observed that 44% of them had both 5hmC peaks at day 20 of differentiation and SOX9 biding peaks (Fig. 3*B*).

To study these potential demethylation dynamics, we built upon our previously published 5hmC maps and profiled 5hmC and 5mC during ATDC5 differentiation at days 0, 10, and 20 (Supplementary Table S2). 5hmC‐containing DNA was enriched and purified using selective chemical conjugation with β‐glucosyltransferase.^(^ ^56^ ^)^ Methylated DNA was enriched based on the MIRA (Active Motif, Inc.) that uses a MBD2b/MBD3L1 protein complex for improved enrichment of CpG dinucleotides^(^ ^34^, ^57^ ^)^ and was subjected to next‐generation sequencing. Significant 5hmC and 5mC peaks were called using the MACs software^(^ ^33^ ^)^ (Supplementary Fig. S2*A–C*). Inspecting *Col2a1* and *Acan*, we observed an increase in 5hmC in ATDC5 chondrocytes at day 10 upon differentiation (compared with day 0) that continued further at day 20 (Fig. 3*C,D*). This 5hmC deposition was near the previously profiled SOX9 binding peaks^(^ ^36^ ^)^ (Fig. 3*C,D*), leading us to hypothesize that SOX9 target genes may globally gain 5hmC during differentiation. To test this hypothesis, we analyzed changes in the 5hmC and 5mC deposition over the TSS and gene bodies of all activated genes (Fig. 3*E,F*) versus SOX9 target genes (Fig. 3*G,H*). Although the activated genes showed the previously described increase in 5hmC (Fig. 3*E*), we observed that this fold increase was more pronounced in the SOX9 target genes (Fig. 3*G*). In contrast, though 5mC was globally increased during chondrogenesis (Fig. 3*F*), we observed that SOX9 target genes were generally hypomethylated at day 0 and accumulated little 5mC with differentiation (Fig. 3*H*). This is consistent with previous observations that showed hypomethylation at SOX9 target genes in chondrocytes.^(^ ^58^ ^)^ Furthermore, we observed that 5hmC peaks in day 20 ATDC5 chondrocytes overlapped, within a 150 bp window (see Materials and Methods section), with 25% of all class II SOX9 peaks, which have been suggested to define the chondrocyte‐specific targets of SOX9,^(^ ^36^ ^)^ as compared with 5% of all class I peaks (Fig. 3*I*). Indeed, 5hmC enrichment along the gene bodies of class II SOX9 genes was greater than along class I genes (Supplementary Fig. S2*D*), and the number of overlapping class II SOX9 peaks increased through differentiation (Supplementary Fig. S2*E*).

To understand what might be unique about this subset of SOX9 genes, we categorized SOX9 targets into two groups: those that had 5hmC peaks in day 20 ATDC5 chondrocytes (6198/9878 or 63%) and those that did not (3680/9878 or 37%). After performing pathway analysis and upstream TF predictions for the 63% of the SOX9 genes with 5hmC, we observed enrichment of TGF‐β regulation of the ECM and NGF, BDNF, and PDGF signaling pathways (Supplementary Fig. S2 *F*). Among the enriched TFs besides SOX9 was WT1, which has been previously shown to recruit TET2 to its binding sites,^(^ ^59^ ^)^ suggesting that TET2 may also contribute to the 5hmC gain during chondrogenesis in ATDC5. RUNX1 has been shown to physically interact with TET2 in the hematopoietic system^(^ ^60^ ^)^ and RUNX2 was another predicted TF, which is known to regulate chondrogenesis alongside SOX9.^(^ ^61^, ^62^, ^63^ ^)^ The RUNX binding motif was also identified using HOMER to predict binding motifs in all the 5hmC reads at day 20 (Supplementary Table S3).

In contrast, among the 37% of SOX9 target genes that did not have 5hmC at day 20 in ATDC5 chondrocytes, we observed enrichment for many cell homeostasis pathways, including cap‐dependent translation, the TCA cycle, and cytoplasmic ribosomal proteins (Supplementary Fig. S2 *G*). Among the enriched TFs were JARID1A, CREB1, ETS1, MYC, and E2F1 (Supplementary Fig. S2 *G*). These observations are consistent with the patterns identified previously, wherein class 1 Sox9 peaks represent tissue‐agnostic binding, whereas class 2 Sox9 peaks are chondrogenic. Collectively, the contrast between these two SOX9 subsets suggests a hypothesis that additional chondrocyte specific factors, such as RUNX2 or TET‐specific partners, such as WT1, may help guide the deposition of 5hmC at this subset of SOX9 genes. Future studies will be needed to elaborate on this possibility.

SOX9 class II sites predominantly overlap with superenhancers^(^ ^36^, ^64^ ^)^; therefore, to further define the SOX9 targets gained 5hmC, we analyzed the overlap between 5hmC peaks in ATDC5 cells and defined rib‐chondrogenic superenhancers.^(^ ^36^ ^)^ We found that the 5hmC gained during differentiation progressively accumulated along superenhancers (Fig. 3*K*).

### *Tet1* knockdown impairs SOX9 binding to *Col2a1* and *Acan*

**Fig 4:**
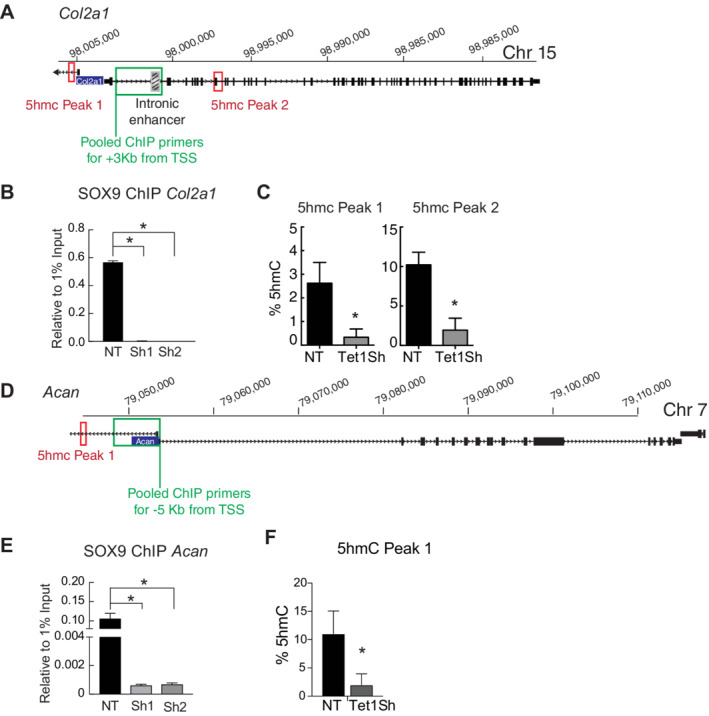
*Tet1* KD impairs SOX9 binding on *Col2a1* and *Acan* in ATDC5. (*A*) Schematic of *Col2a1* and the location of ChIP‐ (chromatin immunoprecipitation‐) qPCR and locus‐specific 5hmC primers. (*B*) ChIP for SOX9 on the *Col2a1* in nontarget (NT) control and two *Tet1Sh* (1 and 2) performed on ATDC5 chondrocytes at day 5. Data are relative to 1% input DNA and represented as mean ± SD (*n* = 1). *p value < 0.05 (Student's *t* test). (*C*) Percentages of 5hmC at a locus‐specific CCGG at two sites on *Col2a1*. Values are expressed as the mean ± SD in *Tet1sh* and NT control (*n* = 2.) *p < 0.05 (Student's *t* test). Data collected at day 5 of ATDC5 differentiation. (*D*) Schematic of *Acan* and the location of ChIP‐qPCR and locus‐specific 5hmC primers. (*E*) ChIP for SOX9 on the *Acan* in NT control and two *Tet1Sh* (1 and 2) performed on ATDC5 chondrocytes at day 5. Data are relative to 1% input DNA and represented as mean ± SD (*n* = 1). *p value <0.05 (Student's *t* test). (*F*) Percentages of 5hmC at a locus‐specific CCGG site on *Acan*. Values are expressed as the mean ± SD in *Tet1sh* and NT control (*n* = 2.) *p < 0.05 (Student's *t* test). Data were collected at day 5 of differentiation.

We next tested if the decreased expression of *Col2a1* and *Acan* in *Tet1* KD cells was based on a loss of SOX9 binding. Previous studies have shown that SOX9 binds to both of these targets, making them good model genes to study the regulation of SOX9 function by TET1.^(^ ^65^, ^66^ ^)^ For this, we performed ChIP for SOX9 early in the ATDC5 differentiation (day 5), when SOX9 is beginning to transactivate its target genes. Using ChIP‐qPCR with a set of pooled primers (Fig. 4*A*), we observed a significant loss of SOX9 binding on *Col2a1* after *Tet1* KD in ATDC5 cells (Fig. 4*B*), thus explaining the loss of *Col2a1* expression. 5hmC loss was also observed after *Tet1* KD in the surrounding CCGG sites (Fig. *4C*). A similar pattern was observed for *Acan* (Fig. 4*D–F*), showing that SOX9 binding was lost in the absence of TET1.

### *Tet1*^*−/−*^ embryos have a mild impairment in the SOX9 target network

**Fig 5:**
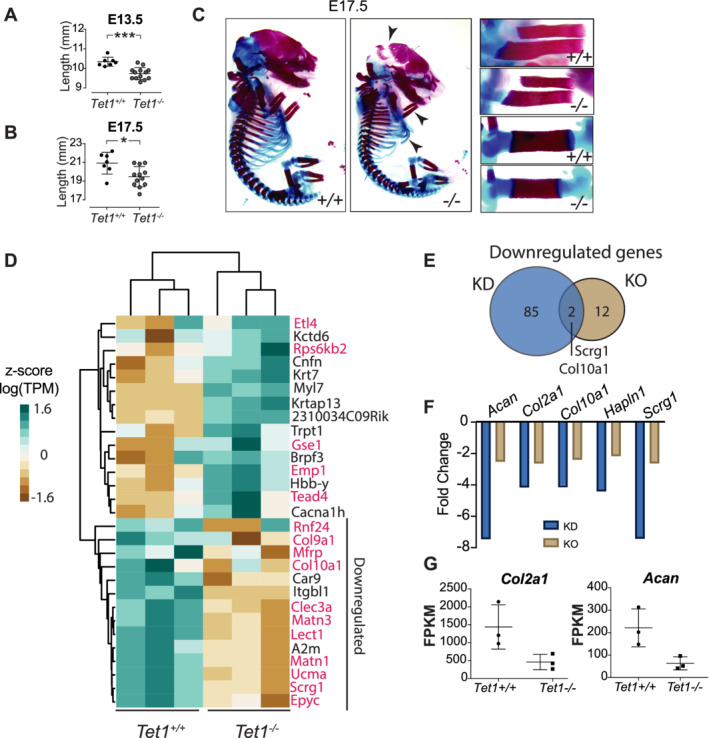
Tet1^−/−^ embryos have a mild impairment in the SOX9 target network. (*A,B*) Quantification of the *Tet1* ^*+/+*^ and *Tet1* ^−/−^ embryos body length (mm) at E13.5 (*n* = 8 *Tet1* ^*+/+*^, *n* = 13 *Tet1* ^*−/−*^) and E17.5 (*n* = 8 *Tet1* ^*+/+*^, *n* = 13 *Tet1* ^*−/−*^). Data are presented as mean ± SD. *p < 0.05, ***p < 0.001 (Student's *t* test). (*C*) Whole‐skeleton staining with Alizarin Red (bone) and Alcian Blue (cartilage) in *Tet1* ^*+/+*^ and *Tet1* ^*−/−*^ littermates at E17.5. Right boxes show higher magnification of the forelimbs (top) and hindlimbs (bottom). Arrows indicate developmental abnormalities. (*D*) Heat map of differentially expressed genes between *Tet1* ^*+/+*^ and *Tet1* ^*−/−*^ growth plates at E17.5. Heat map is clustered based on the *Z*‐score of the log Transcripts Per Million (TPM) for each gene. SOX9 target genes are highlighted in red font. (*E*) Venn diagram of downregulated genes in the knockdown (KD) ATDC5 or the genetic KO. (*F*) Bar graph comparing the fold‐change of key extracellular matrix genes from the KD ATDC5 or the genetic KO. (*G*) FPKM for *Col2a1* and *Acan* in *Tet1* ^*−/−*^ growth plates compared with the *Tet1* ^*+/+*^.

We next sought to investigate the function of TET1 in the growth plate in vivo by analyzing the skeletal development of *Tet1* ^*−/−*^ mice^(^ ^22^ ^)^ and their WT (*Tet1* ^*+/+*^) littermates. We measured body length at embryonic stages E13.5, corresponding to initial chondrogenic differentiation, and E17.5, coinciding with chondrocyte maturation and hypertrophy (Supplementary Fig. S3 *A,B*). At E13.5, the average length of *Tet1* ^*+/+*^ embryos was 10.3 ± 0.2 mm, while *Tet1* ^*−/−*^ embryos measured 9.7 ± 0.3 mm, representing approximately a 6% change in body length (Fig. 5 *A*, Supplementary Table S4). At E17.5, the average length of Tet1^+/+^ embryos was 20.8 ± 1.0 mm, while *Tet1* ^*−/−*^ embryos measured 19.3 ± 1.0 mm, representing approximately a 10% change in body length (Fig. 5 *B*, Supplementary Table S3). To directly compare our data with that of Dawlaty and colleagues, we also recorded the weight of postnatal *Tet1* ^*+/+*^ and *Tet1* ^*−/−*^ mice. Our data are consistent with the previous reports^(^ ^22^, ^28^ ^)^ that there is a disparity in weight between *Tet1* ^*+/+*^ and *Tet1* ^*−/−*^ mice (both male and female) at 3 weeks of age (Supplementary Fig. S3 *C*). This difference disappears in males between 6 to 9 weeks of age, suggesting that the *Tet1* ^*−/−*^ mice catch‐up with *Tet1* ^*+/+*^ controls (Supplementary Fig. S3*C*). We also performed whole‐skeletal staining with Alcian Blue and Alizarin Red of *Tet1* ^*−/−*^ and *Tet1* ^*+/+*^ embryos (Fig. 5*C*), and noted mildly shorter and thinner bones in the trunk and limbs of *Tet1* ^*−/−*^ embryos (Fig. 5*C*, right panels), although overall skeletal development progressed. This mild in vivo phenotype was surprising, given the block in chondrogenic maturation we observed with the *Tet1* KD of ATDC5 progenitors. TET1 expression was observed throughout the growth plate and was absent from *Tet1* ^*−/−*^ embryos (Supplementary Fig. S3 *D*).

To understand the functional implication of TET1 loss, we performed RNA sequencing on chondrocytes isolated from the growth plates of E17.5 *Tet1* ^*−/−*^ and *Tet1* ^*+/+*^ growth plates. Cartilage from E17.5 hind limbs was dissected and snap‐frozen in liquid nitrogen prior to RNA extraction. Differential gene expression analyses revealed that, in contrast to the large number of genes altered in ATDC5 cells upon *Tet1* loss, only 14 and 15 genes were significantly down‐ and upregulated, respectively, in the *Tet1* ^*−/−*^ growth plate (Fig. 5*D*). However, 12 out of the 14 genes (85%) that were downregulated 2.5‐fold or greater, are SOX9 targets^(^ ^36^ ^)^ (Fig. 5*D*). Two of these targets, *Scrg1* and *Col10a1*, are shared TET1 targets from ATDC5 (Fig. 5*E*). When we compared the fold‐change of the genes significantly affected in ATDC5 upon *Tet1* KD in the *Tet1* ^*−/−*^ embryos, we observed that most were downregulated, but more modestly than in ATDC5 (Fig. 5*F*). For example, *Acan* was downregulated almost eightfold in *Tet1* KD ATDC5 cells, but only a twofold reduction was observed in the *Tet1* ^*−/−*^ growth plate. Similarly, *Col2a1* is not greatly affected, although there is a downward trend (Fig. 5*G*). The difference in the magnitude of gene expression reflects the discrepancy between the in vitro and in vivo phenotypes. Similar to the ATDC5 cells, no loss in *Sox9* mRNA was observed in the *Tet1* ^*−/−*^ growth plate as shown by the RNA‐sequencing data and by additional validation by qPCR on growth plate cartilage tissue (Supplementary Fig. S3 *E*). We also isolated a highly pure population of chondrocytes from E17.5 embryos (Supplementary Fig. S3 *F*), further expanded for a limited number of passages (1 to 2), and observed similar protein levels for Sox9 from *Tet1* ^*−/−*^ and *Tet1* ^*+/+*^ chondrocytes (Supplementary Fig. S3 *G*). This modest alteration in the transcriptome suggests that additional mechanisms are at play in vivo, with one possible explanation being functional compensation by TET2 and/or TET3. Upon testing, their expression levels were found to be unchanged in the *Tet1* ^*−/−*^ growth plate (Supplementary Fig. S3 *H*).

### TET1 loss in growth plates changes the 5hmC landscape

To better understand the 5hmC landscape in the *Tet1* ^*+/+*^ and *Tet1* ^*−/−*^ embryos, we profiled global 5hmC in E17.5 chondrocytes in the *Tet1* ^*+/+*^ and *Tet1* ^*−/−*^ embryos. A modest loss of 5hmC was observed via immunofluorescence (Supplementary Fig. S4 *A,B*), highlighting that only part of the global 5hmC landscape is specific to TET1 function in vivo. To assess which specific genes were losing 5hmC, and how this compared with the 5hmC observed in ATDC5 cells, we performed genomewide sequencing. Because of the scarcity of tissue material from cartilage microdissected from E17.5 embryos (see Materials and Methods section), we utilized a RRHP of CCGG sites,^(^ ^46^, ^47^ ^)^ which allowed us to probe the 5hmC status of a select number of sites in the mouse genome (Supplementary Table S5).

**Fig 6:**
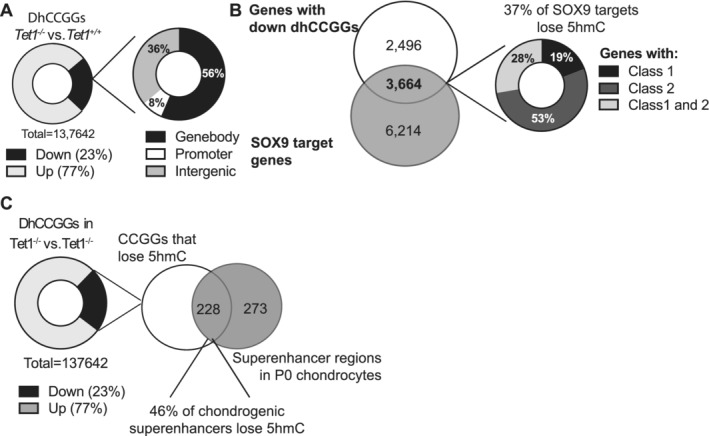
*Tet1* loss in the growth plates changes the 5hmC landscape. (*A*) Pie chart of differentially hydroxymethylated CCGG sites between *Tet1* ^*−/−*^ and *Tet1* ^*+/+*^ growth plates (*n* = 3 for each genotype). Genes with dhCCGGs losing 5hmC were overlapped and classified as either being in the gene body (between TSS and TTS), promoter (up to 5000 bp from TSS) or intergenic, as shown in the pie chart on the right. (*B*) Venn diagram of genes with dhCCGGs losing 5hmC with genes containing SOX9 binding sites. The overlapping genes were then classified as either containing class I, class II, or both types of binding sites and the percentage of each was tabulated in the pie chart on the right. Overall, overlapping genes represent approximately 10% of class I and class II Sox9 sites. (*C*) Pie chart of the number of dCCGGs losing 5hmC that overlap with superenhancer regions in P0 chondrocytes.

We observed that although multiple CCGG sites lost 5hmC in the absence of TET1, there were also many sites that gained 5hmC (Fig. 6*A*). This accumulation of 5hmC may be from loss of further 5hmC oxidation (ie, 5caC and 5fC), or from increased deposition by TET2 and TET3. The majority (56%) of the 5hmC loss was from gene bodies (Fig. 6*A*), consistent with the 5hmC buildup that was primarily observed in the gene bodies in ATDC5 cells, whereas 37% of SOX9 targets lost 5hmC peaks in the *Tet1* ^*−/−*^ embryos, with the majority of these being from the chondrocyte‐specific class 2 peaks (Fig. 6*B*). However, overall approximately only 10% and 9% of all class I and class II SOX9 binding sites, respectively, showed 5hmC loss. Similar to the ATDC5 data, we also observed a loss of 5hmC from superenhancers (Fig. 6*C*). Analysis of 5hmC at 4 CCGG sites on *Acan* and two CCGG sites on *Col2a1* revealed that 5hmC was lost from only a few CCGG sites, while others sites actually gained 5hmC in these genes (Supplementary Fig. S4 *C*), consistent with the modest and insignificant changes in their expression as compared with the ATDC5 cells.

## Discussion

The TET enzymes, and the cytosine modifications they impart, are critical in development and tissue differentiation.^(^ ^12^, ^13^, ^14^ ^)^ In this study, we investigated the role of TET1 in the context of chondrogenesis. We found that in ATDC5 chondroprogenitor cells, shRNA KD of *Tet1* resulted in a block in differentiation, characterized by a loss of GAG deposition and loss of *Col2a1*, *Acan*, and other ECM gene expression. Transcriptome analysis demonstrated a loss of activation of a subset of the SOX9 transcriptional network. Among the 90 genes that were downregulated in the *Tet1* KD cells, 54% were SOX9 targets, despite no observed change in expression of *Sox9* or its known common binding partners.

Previous work on the relationship between TET1 and SOX9 in ISCs showed that TET1‐mediated 5hmC deposition on the *Sox9* promoter regulates its expression.^(^ ^55^ ^)^ We did not observe a similar transcriptional regulation of *Sox9* at the mRNA or protein level, and KD of *Tet1* did not result in loss of 5hmC at two previously defined peaks in *Sox9*. Thus in chondrocytes, in contrast to ISCs, TET1‐mediated 5hmC does not appear to regulate *Sox9* expression. It remains possible, however, that *Tet1* KD maybe have other indirect effects on SOX9 protein function in the ATDC5 system, including changes to splicing and posttranslational modifications of SOX9 or of its partner proteins, which were not assayed here.

Regardless of the way the SOX9 complex is disrupted, we observed that *Tet1* KD resulted in a loss of SOX9 binding at two key lineage genes, *Col2a1* and *Acan*, early in ATDC5 differentiation (day 5) when lineage genes are first activated. At the same time point, 5hmC is lost in the surrounding area, suggesting that the presence of TET1‐mediated 5hmC is a requirement prior to SOX9 binding. A recent study, where *Sox9* was conditionally deleted from the precondensed mesenchyme,^(^ ^67^ ^)^ elegantly demonstrated that the epigenome was not altered by the loss of *Sox9*, corroborating the hypothesis that the epigenome conducive to the chondrogenic lineage is set preceding SOX9 binding. We hypothesized that 5hmC deposition precedes and facilitates SOX9 binding; however, our experiments could not differentiate whether TET1 affects SOX9 binding directly or in an indirect fashion by affecting one of its binding partners. Additionally, as shown by our temporal global data, 5hmC is continually built over ATDC5 differentiation, thus a more complex and dynamic interplay between SOX9 and TET1 likely exists, with both of them facilitating each otherʼs functions.

In trying to understand the relationship between SOX9 and 5hmC, we observed that 5hmC is preferentially enriched in the class II SOX9 sites that are chondrocyte specific as opposed to the tissue agnostic class I binding sites. Distinct binding mechanisms have been suggested for the binding of SOX9 to class I and II sites.^(^ ^36^ ^)^ Although SOX9 has a higher affinity but indirect binding to class I sites, its binding to class II sites is direct but low affinity, necessitating cooperativity with itself or partner proteins. This low‐affinity binding may be facilitated by the presence of 5hmC that has been associated with increased DNA flexibility and accessibility.^(^ ^68^, ^69^ ^)^ Although 63% of all SOX9 target genes have 5hmC marks, only 25% of the SOX9 class II peaks present in these genes overlap with 5hmC within a 150‐bp window. This suggests that 5hmC deposition is associated with a particular mode of SOX9 binding, rather than a general requirement for all SOX9 sites. Because this subset is highly enriched in chondrogenic superenhancers, one hypothesis is that 5hmC‐deposition facilitates the binding or formation of specific SOX9 complexes.

The comparison between our in vitro acute KD system and our in vivo genetic KO model mirrors the discrepancy between *Tet1* KD and KO in mouse ESCs, in which KD^(^ ^70^ ^)^ results in a greater loss of pluripotency‐associated genes than KO.^(^ ^22^ ^)^ Likewise, we see a differentiation block after *Tet1* KD, which is not fully recapitulated in the global KO mouse model. These data suggest the hypothesis that during embryonic development, the TET paralogues may rewire themselves and compensate for the missing family member, albeit not completely.^(^ ^71^ ^)^ Future work will be needed to test whether such a rewiring occurs in chondrogenesis, as well upon genetic loss of TET1. TET2 and TET3 have already been shown to be important in adult mesenchymal stem cell (MSC) differentiation^(^ ^72^, ^73^, ^74^ ^)^; however, their unique contributions vis‐à‐vis TET1 remain to be explored. Additionally, in the KD of *Tet1*, we observed that 72% of differentially expressed genes were upregulated. This likely reflects TET1ʼs role as a repressor via its interaction with the PRC2 complex.^(^ ^14^, ^52^ ^)^ Further studies should elucidate the roles of this repressive function of TET1, which has already been described in human MSCs,^(^ ^72^ ^)^ in growth plate development.

Collectively, these data provide insights about SOX9 function in chondrogenesis by establishing a role for TET1 in the expression of a subset of SOX9 target genes during ATDC5 differentiation. Future studies will be required to elucidate the precise cofactors that contribute to TET1‐mediated 5hmC and SOX9 binding, and test whether 5hmC deposition by TET 2 or 3 also plays a role.

## Disclosures

The authors have no financial interests to disclose.

The peer review history for this article is available at https://publons.com/publon/10.1002/jbm4.10383.

## Supporting information


**Supplemental Figure S1** (related to Figs. 1 and 2): (*A*) qPCR gene expression analysis for *Tet2* and *Tet3* in NT control and *Tet1sh* ATDC5 chondrocytes. Expression is normalized to *Gapdh* and fold change is relative to non‐target. Data are represented as mean ± SD (*n* = 3). (*B*) Dot Blot analysis for global 5hmC and 5mC levels during ATDC5 chondrogenic differentiation (day 0, day 5, day 10, day 15) in the presence (non‐target) or absence (Sh1, Sh2 and Sh3) of TET1. (*C*) Biopathways associated with genes whose expression is increased after *Tet1KD* in day 15 ATDC5 cells. (*D*) Percent of genes whose expression is increased at *Tet1KD* which have SOX9 peaks and 5hmc peaks. (*E*) RNA‐sequencing analysis of *Sox9* and *Tet1* in human embryonic stem cell (hESC) differentiation to mesoderm through the intermediate stages of anterior primitive streak (APS), paraxial mesoderm (PXM), somite (SOM) and sclerotome formation (Sclerot). Data reanalyzed from.^(^ ^76^ ^)^ (*F*) Real‐time PCR gene expression analysis of *Tet1* and *Sox9* in NT control and *Tet1sh* ATDC5 chondrocytes at day 0 (before differentiation) Expression is normalized to *Gapdh* and fold change is relative to one of the NT controls. Data are represented as mean ± SD of three independent biological replicates (n = 3).ns = not significant, ** is *p*‐value =0.004 (one‐way ANOVA with multiple comparisons).

**Supplemental Figure S2** (related to Fig. 3): (*A*) Saturation plots for the unique BWA aligned reads for 5mC sequencing showing high correlation and good agreement between the estimated and actual saturation curves for all samples. (*B,C*) Distribution of 5hmC and 5mC in individual genomic compartments in the progenitor, intermediate and chondrocytes. 5mC and 5hmC peaks observed in the particular compartment are depicted as a percentage of the total 5hmC or 5mC peaks, respectively, for each time point. (*D*) Composite profiles of the 5hmC distribution on SOX9 Class I or Class II genes during differentiation from progenitor (day 0), intermediate (day 10), and chondrocyte (day 20) averaged over 5′‐3′ interval length ± 1000 bp. (*E*) Percent of SOX9 peaks (either Class 1 or Class 2) which overlap with 5hmC peaks profiled at days 0, 10 and 20 of ATDC5 differentiation. (*F*) Biopathways, generated using Enrichr, of the SOX9 target genes that overlap with 5hmC at day 20. (g) Biopathways and upstream TFs of the SOX9 target genes that do not overlap with 5hmC peaks at day 20 in ATDC5 cells.

**Supplemental Figure S3** (related to Fig. 5): (*A*) Representative gross appearance of a *Tet1* ^*−/−*^ embryo at E17.5 compared to the *Tet1* ^*+/+*^ control littermate. Black arrows designate the body length. (*B*) Table of *Tet1* ^*+/+*^ and *Tet1* ^−/−^ embryos showing a smaller body ratio. *p value <0.05 (Student's t test). (*C*) Body weight of each genotype at postnatal week 3, or 6–9. Last two graphs represent the breakdown weight by sex (male vs. female). (*D*) Immunostaining for TET1 protein (red) in *Tet1* ^*+/+*^ and *Tet1* ^*−/−*^ femur growth plates at E17.5. Nuclei are counterstained with DAPI (blue). (*E*) Real‐time PCR of *Sox9* expression in *Tet1* ^*+/+*^ and *Tet1* ^*−/−*^ cartilage tissue at E17.5. Expression is normalized to *Gapdh* and fold change is relative to one of the *Tet1* ^*+/+*^ embryos. Data are represented as the mean ± SD. *n* = 4 from each genotype. (*F*) Representative FACS analysis (*n* = 2) to assess purity of primary mouse chondrocytes isolated from the growth plate at E17.5, cultured and stained with CD200‐PerCP, CD24‐FITC and SOX9‐PE antibodies. Cells are gated on the CD200 and CD24 expression (top panels) and they are then analyzed for SOX9 expression (bottom panels). (*G*) Representative Western blot for SOX9 and GAPDH protein levels in primary mouse chondrocytes from *Tet1* ^*+/+*^ and *Tet1* ^*−/−*^ growth plates at E17.5. SOX9 levels are normalized to GAPDH and quantified by ImageJ (histogram on the bottom). Data are presented as mean ± SD (n = 2). (*H*) Gene expression analysis for *Tet1, Tet2 and Tet3* in *Tet1* ^*+/+*^ and *Tet1* ^*−/−*^ cartilage tissue at E17.5. Expression is normalized to *Gapdh* and fold change is relative to the expression of *Tet1* in *Tet1* ^*+/+*^cells. Data are represented as mean ± SD (*n* = 5).

**Supplemental Figure S4** (related to Fig. 6): (*A*) Immunostaining for 5hmC (red) in *Tet1* ^*+/+*^ and *Tet1* ^*−/−*^ growth plate at E17.5. Nuclei are counterstained with DAPI (blue). (*B*) Quantification of two images for each pup shown in panel A using Fiji. Means compared using student's t‐test (*p‐value = 0.05) (*C*) Individual CCGG sites from the RRHP analysis plotted for *Acan* and *Col2a1* from *Tet1* ^*+/+*^ and *Tet1* ^*−/−*^ growth plate. Data is represented as the fold change of the *Tet1* ^*+/+*^ (n = 3) to the *Tet1* ^*−/−*^ embryos (*n* = 3). Only CCGG sites that tested significant after global multiple hypothesis correction are plotted.

**Supplemental Table S1** Differentially expressed genes from RNA‐seq and microarray analysis.

**Supplemental Table S2** Location of 5hmC and 5mC peaks from ATDC5 differentiation.

**Supplemental Table S3** HOMER analysis of 5hmC peaks in ATDC5

**Supplemental Table S4** Embryo measurements at E13.5 and 17.5

**Supplemental Table S5** Location of differentially hydroxymethylated CCGG sites.
