# Supplementary figures and images for: TET1 Directs Chondrogenic Differentiation by Regulating SOX9 Dependent Activation of Col2a1 and Acan In Vitro
Source: JBMR Plus. 2020 Jun 26;4(8):e10383. doi: 10.1002/jbm4.10383 (PMC7587462; doi:10.1002/jbm4.10383)

Figure S1 (related to Figure 1&2):

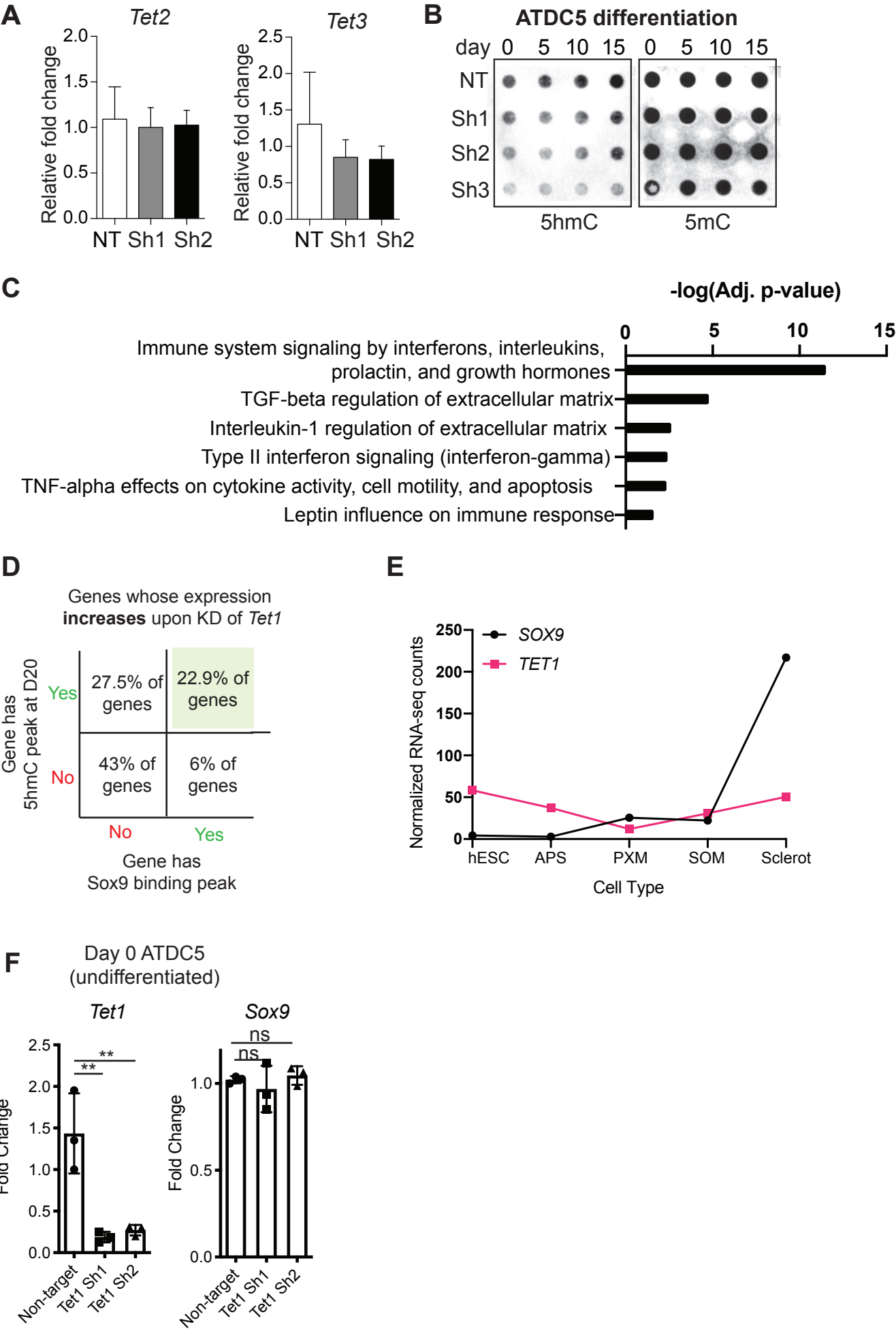

Supplement: Supplementary file 1 — Supplemental Figure S1 (related to Figs. 1 and 2): (A) qPCR gene expression analysis for Tet2 and Tet3 in NT control and Tet1sh ATDC5 chondrocytes. Expression is normalized to Gapdh and fold change is relative to non‐target. Data are represented as mean ± SD (n = 3). (B) Dot Blot analysis for global 5hmC and 5mC levels during ATDC5 chondrogenic differentiation (day 0, day 5, day 10, day 15) in the presence (non‐target) or absence (Sh1, Sh2 and Sh3) of TET1. (C) Biopathways associated with genes whose expression is increased after Tet1KD in day 15 ATDC5 cells. (D) Percent of genes whose expression is increased at Tet1KD which have SOX9 peaks and 5hmc peaks. (E) RNA‐sequencing analysis of Sox9 and Tet1 in human embryonic stem cell (hESC) differentiation to mesoderm through the intermediate stages of anterior primitive streak (APS), paraxial mesoderm (PXM), somite (SOM) and sclerotome formation (Sclerot). Data reanalyzed from.( 76 ) (F) Real‐time PCR gene expression analysis of Tet1 and Sox9 in NT control and Tet1sh ATDC5 chondrocytes at day 0 (before differentiation) Expression is normalized to Gapdh and fold change is relative to one of the NT controls. Data are represented as mean ± SD of three independent biological replicates (n = 3).ns = not significant, ** is p‐value =0.004 (one‐way ANOVA with multiple comparisons). [file JBM4-4-e10383-s001.pdf]

**Figure S2 (related to Figure 3):**

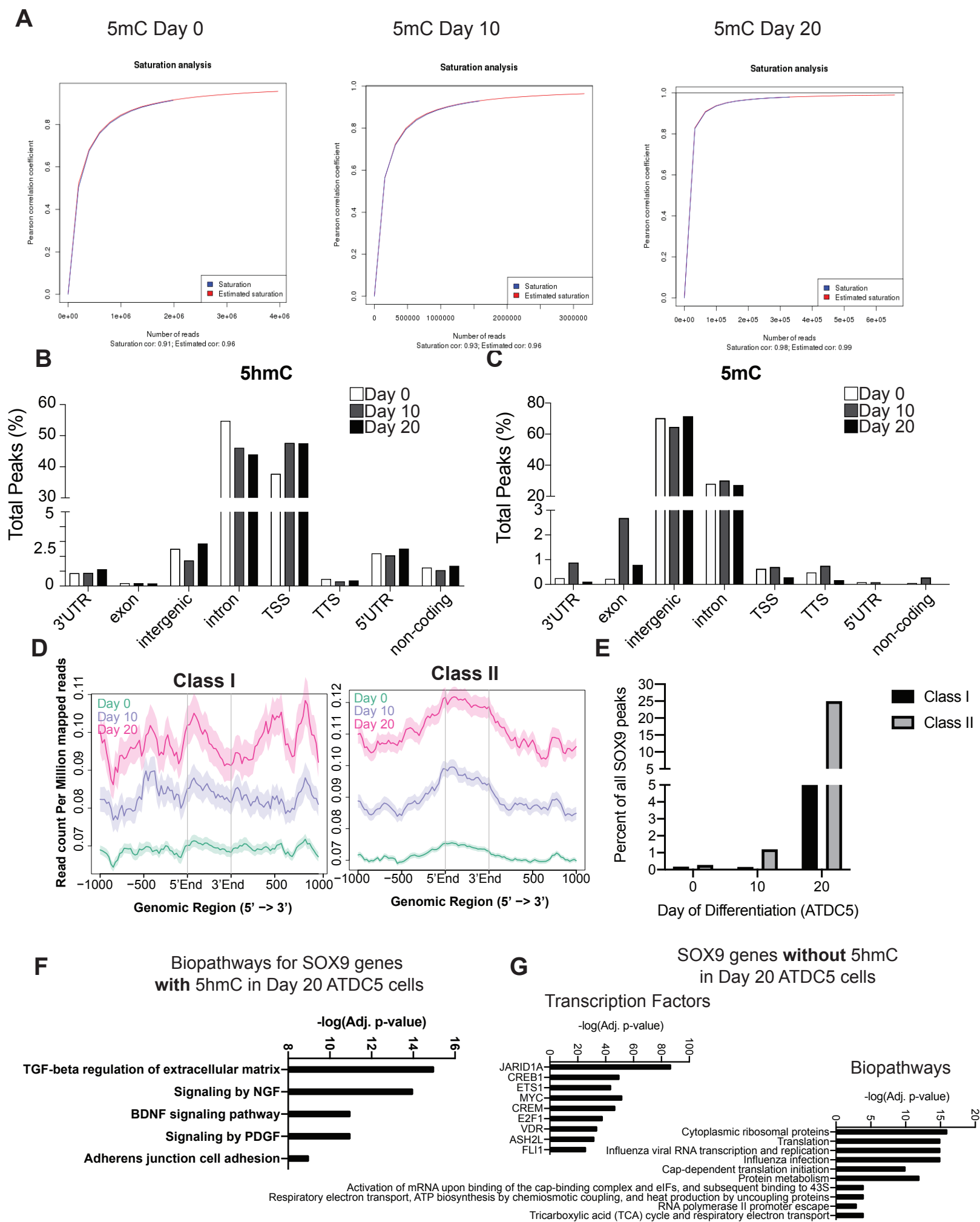

Supplement: Supplementary file 2 — Supplemental Figure S2 (related to Fig. 3): (A) Saturation plots for the unique BWA aligned reads for 5mC sequencing showing high correlation and good agreement between the estimated and actual saturation curves for all samples. (B,C) Distribution of 5hmC and 5mC in individual genomic compartments in the progenitor, intermediate and chondrocytes. 5mC and 5hmC peaks observed in the particular compartment are depicted as a percentage of the total 5hmC or 5mC peaks, respectively, for each time point. (D) Composite profiles of the 5hmC distribution on SOX9 Class I or Class II genes during differentiation from progenitor (day 0), intermediate (day 10), and chondrocyte (day 20) averaged over 5′‐3′ interval length ± 1000 bp. (E) Percent of SOX9 peaks (either Class 1 or Class 2) which overlap with 5hmC peaks profiled at days 0, 10 and 20 of ATDC5 differentiation. (F) Biopathways, generated using Enrichr, of the SOX9 target genes that overlap with 5hmC at day 20. (g) Biopathways and upstream TFs of the SOX9 target genes that do not overlap with 5hmC peaks at day 20 in ATDC5 cells. [file JBM4-4-e10383-s002.pdf]

**Figure S3 (related to Figure 5).**

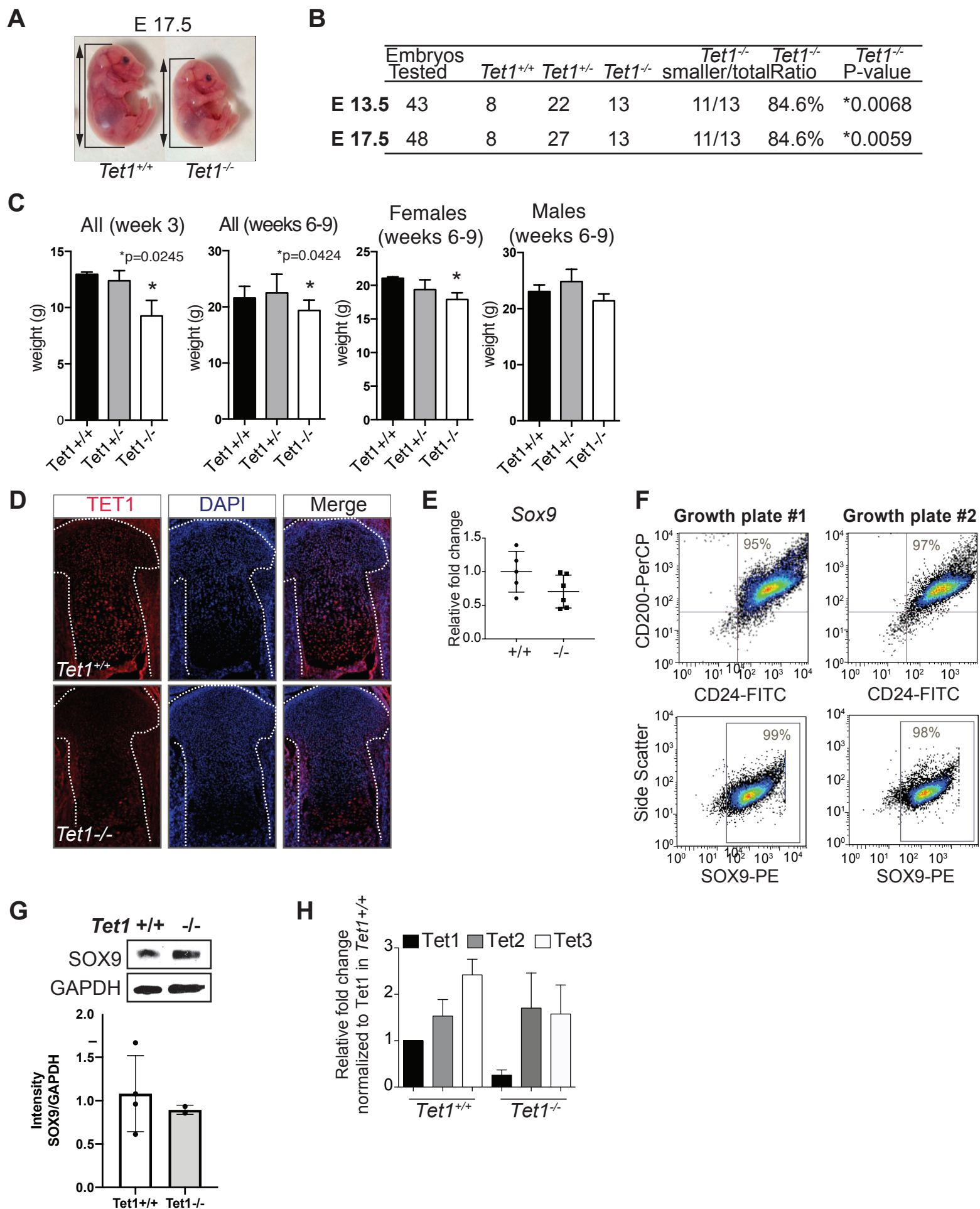

Supplement: Supplementary file 3 — Supplemental Figure S3 (related to Fig. 5): (A) Representative gross appearance of a Tet1 −/− embryo at E17.5 compared to the Tet1 +/+ control littermate. Black arrows designate the body length. (B) Table of Tet1 +/+ and Tet1 −/− embryos showing a smaller body ratio. *p value <0.05 (Student's t test). (C) Body weight of each genotype at postnatal week 3, or 6–9. Last two graphs represent the breakdown weight by sex (male vs. female). (D) Immunostaining for TET1 protein (red) in Tet1 +/+ and Tet1 −/− femur growth plates at E17.5. Nuclei are counterstained with DAPI (blue). (E) Real‐time PCR of Sox9 expression in Tet1 +/+ and Tet1 −/− cartilage tissue at E17.5. Expression is normalized to Gapdh and fold change is relative to one of the Tet1 +/+ embryos. Data are represented as the mean ± SD. n = 4 from each genotype. (F) Representative FACS analysis (n = 2) to assess purity of primary mouse chondrocytes isolated from the growth plate at E17.5, cultured and stained with CD200‐PerCP, CD24‐FITC and SOX9‐PE antibodies. Cells are gated on the CD200 and CD24 expression (top panels) and they are then analyzed for SOX9 expression (bottom panels). (G) Representative Western blot for SOX9 and GAPDH protein levels in primary mouse chondrocytes from Tet1 +/+ and Tet1 −/− growth plates at E17.5. SOX9 levels are normalized to GAPDH and quantified by ImageJ (histogram on the bottom). Data are presented as mean ± SD (n = 2). (H) Gene expression analysis for Tet1, Tet2 and Tet3 in Tet1 +/+ and Tet1 −/− cartilage tissue at E17.5. Expression is normalized to Gapdh and fold change is relative to the expression of Tet1 in Tet1 +/+cells. Data are represented as mean ± SD (n = 5). [file JBM4-4-e10383-s003.pdf]

Figure S4 (related to Figure 6).

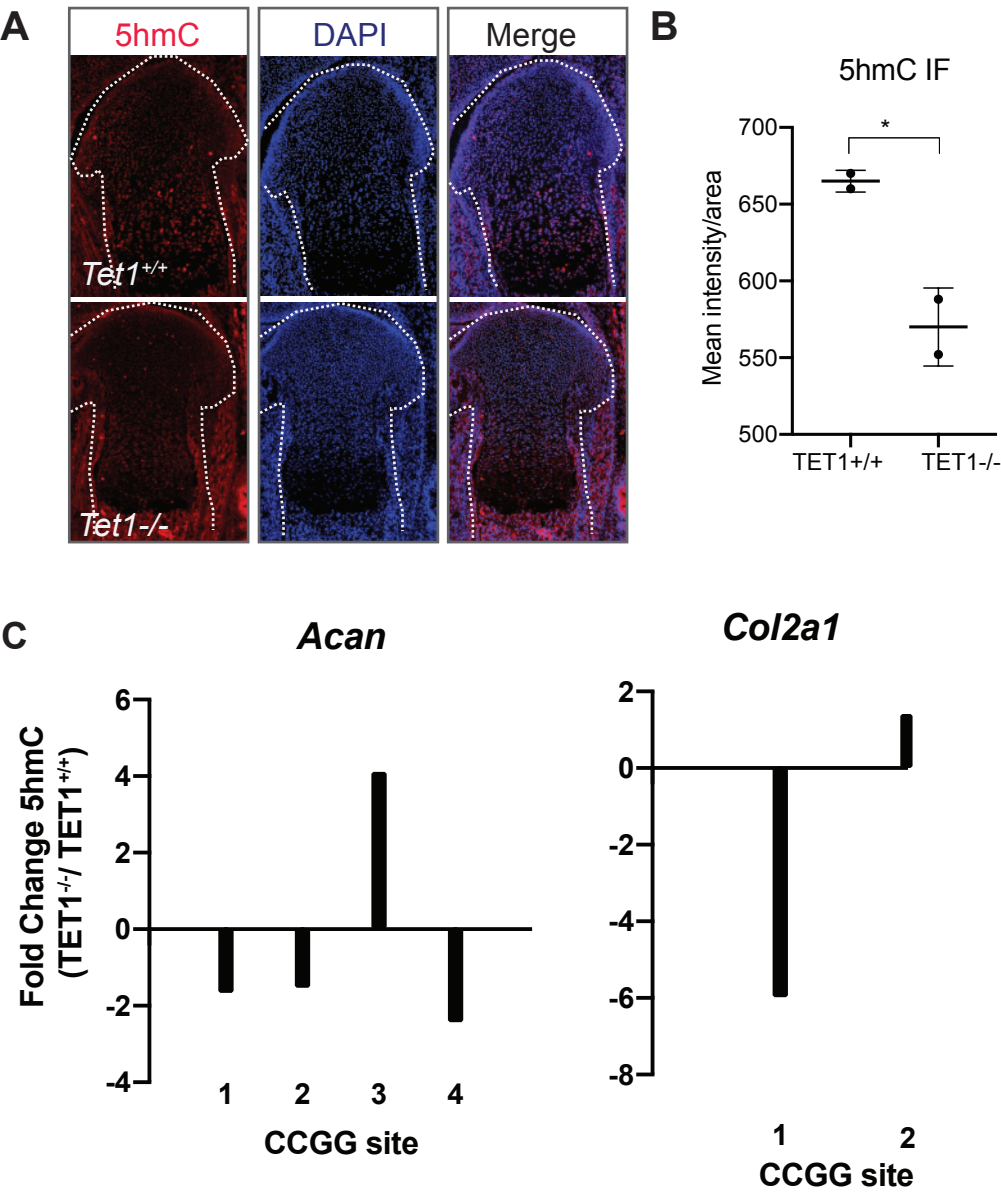

Supplement: Supplementary file 4 — Supplemental Figure S4 (related to Fig. 6): (A) Immunostaining for 5hmC (red) in Tet1 +/+ and Tet1 −/− growth plate at E17.5. Nuclei are counterstained with DAPI (blue). (B) Quantification of two images for each pup shown in panel A using Fiji. Means compared using student's t‐test (*p‐value = 0.05) (C) Individual CCGG sites from the RRHP analysis plotted for Acan and Col2a1 from Tet1 +/+ and Tet1 −/− growth plate. Data is represented as the fold change of the Tet1 +/+ (n = 3) to the Tet1 −/− embryos (n = 3). Only CCGG sites that tested significant after global multiple hypothesis correction are plotted. [file JBM4-4-e10383-s004.pdf]
